# Associations of Central Auditory Processing With Brain Volumes

**DOI:** 10.1093/geroni/igab046.597

**Published:** 2021-12-17

**Authors:** Kening Jiang, Josef Coresh, Kathleen Hayden, Clifford Jack, Thomas Mosley, James Pankow, Frank Lin, Jennifer Deal

**Affiliations:** 1 Johns Hopkins Cochlear Center for Hearing and Public Health, Baltimore, Maryland, United States; 2 Department of Epidemiology, Johns Hopkins Bloomberg School of Public Health, Baltimore, Maryland, United States; 3 Wake Forest School of Medicine, Winston-Salem, North Carolina, United States; 4 Department of Radiology, Mayo Clinic, Rochester, Minnesota, United States; 5 The University of Mississippi Medical Center, Jackson, Mississippi, United States; 6 Division of Epidemiology and Community Health, University of Minnesota School of Public Health, Minneapolis, Minnesota, United States; 7 Johns Hopkins University, Johns Hopkins University, Maryland, United States; 8 Johns Hopkins University, Baltimore, Maryland, United States

## Abstract

We investigated the cross-sectional associations of speech-in-noise performance with magnetic resonance imaging brain volumes among 588 cognitively normal participants (77±4 years, 56% female) from the Aging and Cognitive Health Evaluation in Elders Study (randomized trial embedded in the Atherosclerosis Risk in Communities (ARIC) Study) baseline in 2018-19 (N=427, with hearing loss) and ARIC (N=161, normal hearing) Visit 6/7 in 2016-17/2018-19. Central auditory processing was measured by Quick Speech-in-Noise (QuickSIN) test; range: 0 to 30, lower scores=worse performance. In models adjusted for demographic and disease covariates, every 5-point decrease in QuickSIN score was associated with smaller volumes of the temporal lobe overall (-0.07SD, 95% CI:-0.13,-0.01) as well as subregions including but not limited to those important for auditory processing (amygdala:-0.13SD, 95% CI:-0.21,-0.04; middle temporal gyrus:-0.08SD, 95% CI:-0.15,-0.00; superior temporal gyrus:-0.08SD, 95% CI:-0.15,-0.01). Further research is needed to understand the mechanisms underlying these observed associations.

